# Supplementary material for: Integrated time course omics analysis distinguishes immediate therapeutic response from acquired resistance
Source: Genome Med. 2018 May 23;10:37. doi: 10.1186/s13073-018-0545-2 (PMC5966898; doi:10.1186/s13073-018-0545-2)

Figure S1 - Time course approach to induce resistance to cetuximab and measure gene expression and DNA methylation changes. Intrinsic cetuximab sensitive HNSCC cell line SCC25 were treated with cetuximab (red) or PBS (black) for 7 days. In the eighth day, cells were collected and pooled from multiple replicate cultures to provide adequate amounts for total RNA isolation for RNA-seq, genomic DNA isolation for DNA methylation array, proliferation assay (flow), for storage (frozen) and to be plated again to continue treatment until resistance to cetuximab developed. Each collection point was called a generation (from CTX-G0 to CTX-G11).

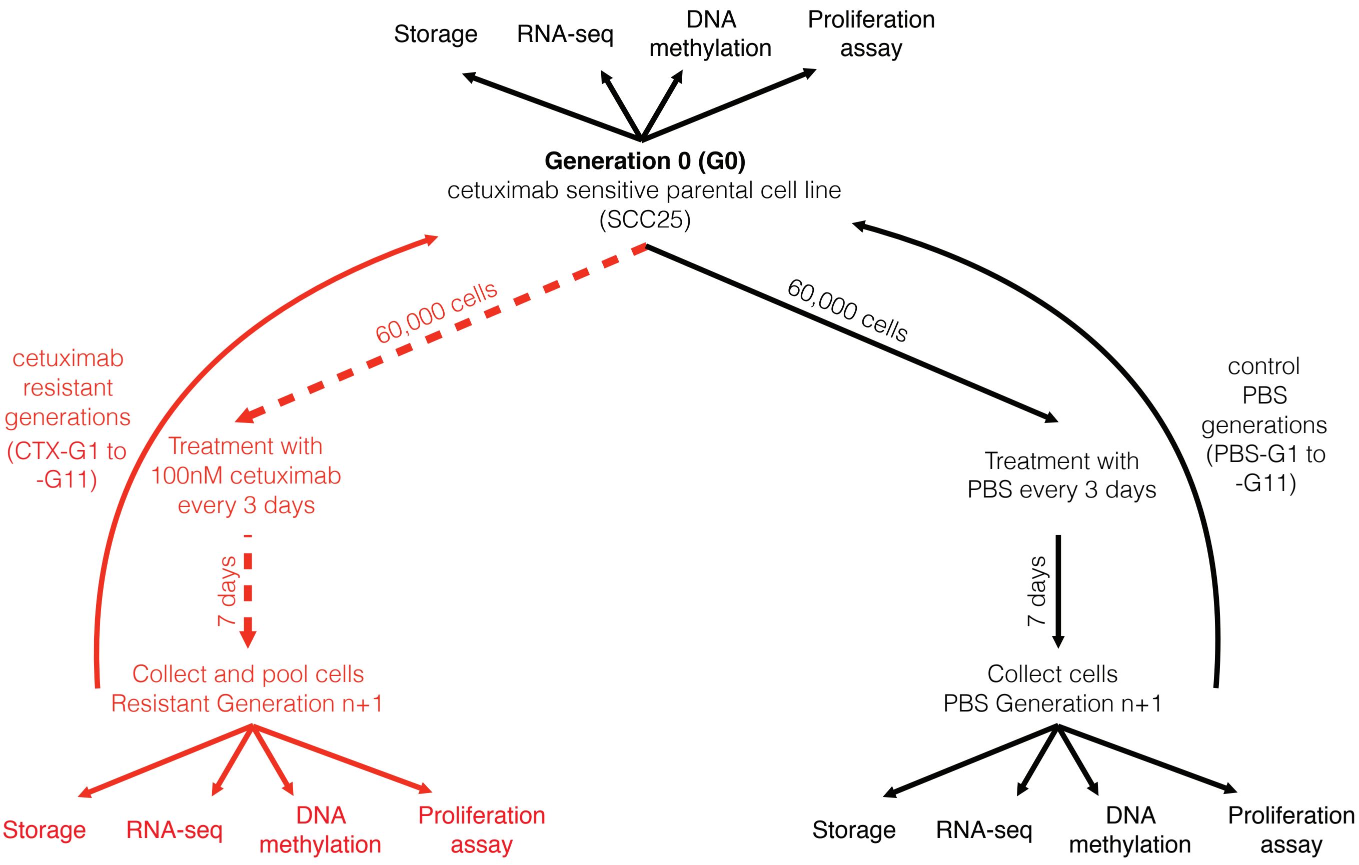

Figure S2 - Anchorage-independent growth of cetuximab generation 10 (CTX-G10). Colony formation assay in matrigel to confirm acquired cetuximab resistance of CTX-G10 (red) relative to the parental cell line (CTX-G0, black) at different concentrations of cetuximab (0nM, 10nM, 100nM and 1000nM).

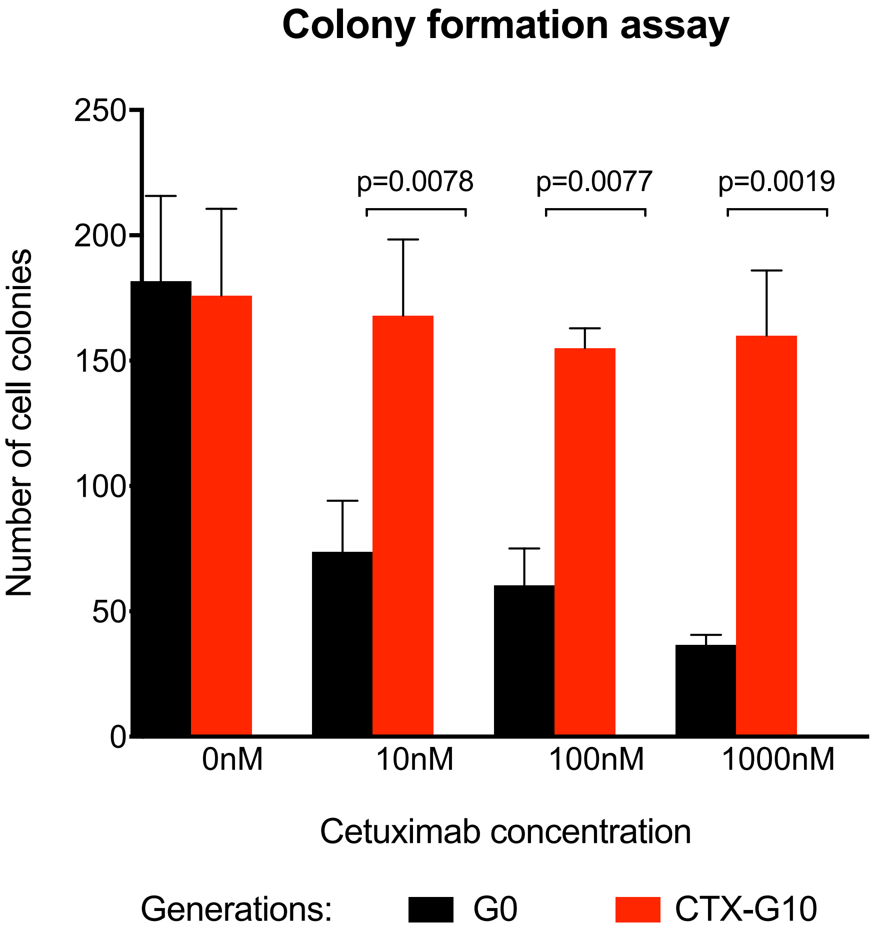

Figure S3 - Heatmap and hierarchical clustering of gene expression values in 11 generations of SCC25 cells treated with PBS as control (black columns) and with 100nM of cetuximab (red columns) to acquire resistance.

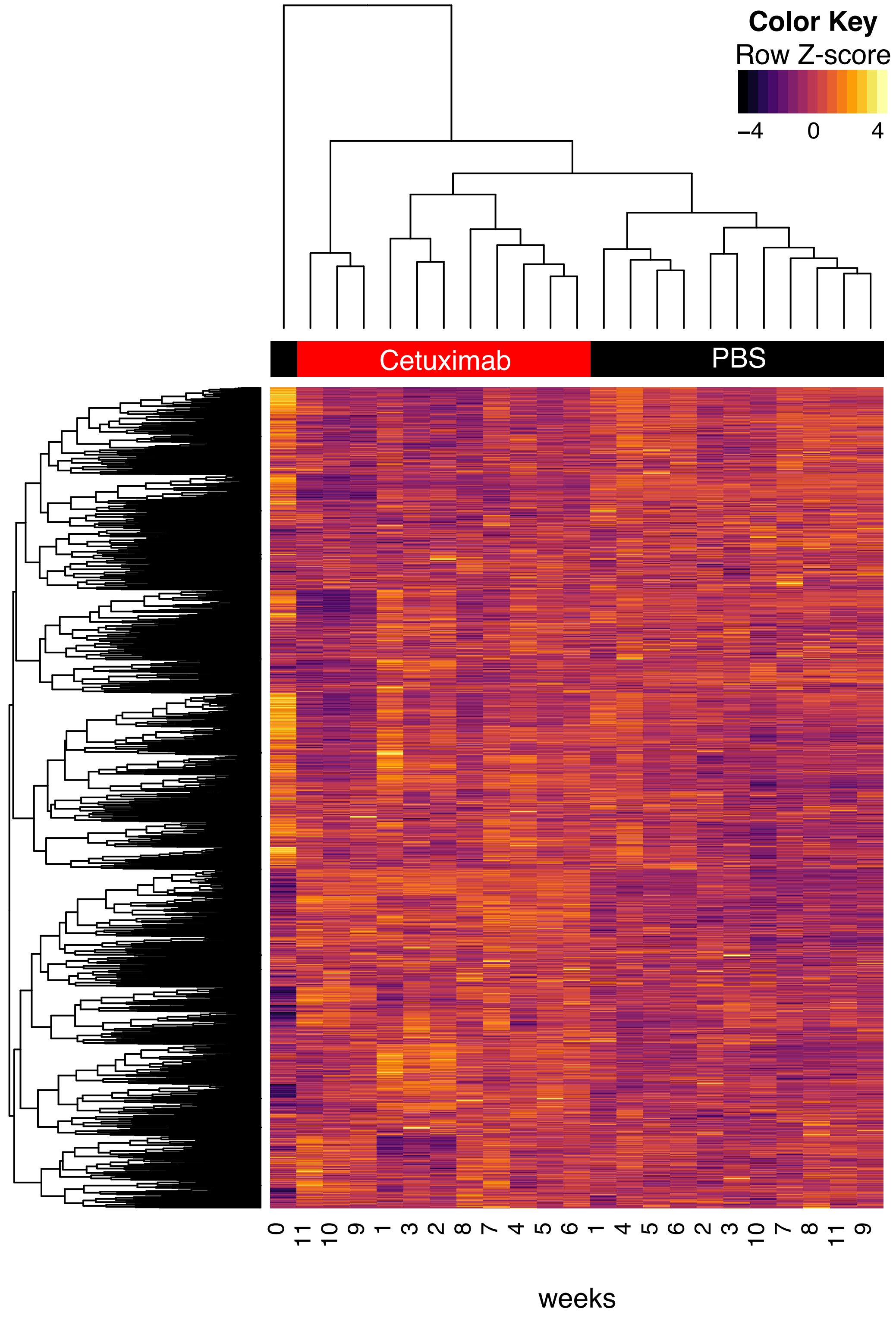

Figure S4 - Time course gene expression compared to previously known gene signatures of resistance to EGFR inhibitors. A. Heatmap of gene expression values in 11 generations of SCC25 cells treated with 100nM of cetuximab (red columns) to acquire resistance and with PBS as control (black columns). Genes selected for visualization were associated with cetuximab resistance from previous gene expression studies comparing sensitive and resistant cells without regard for timing. These studies provided three gene sets, colored along rows of the heatmap. B. Average of z-score gene expression values for genes in each of the resistance signatures over generations of PBS control (black lines) or treatment with 100nM of cetuximab (red lines).

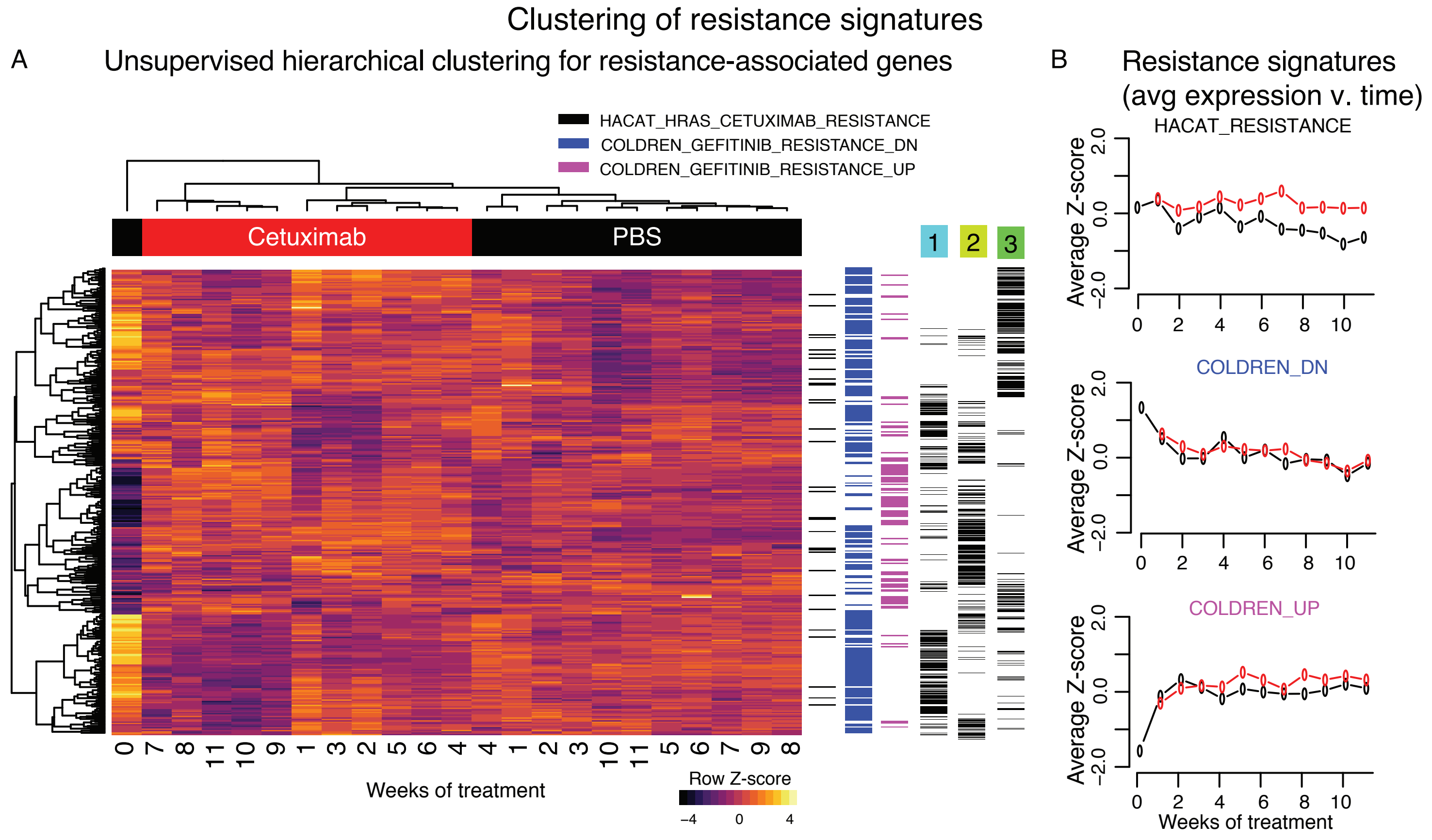

Figure S5 - Expected gene expression values for genes in each CoGAPS pattern inferred from gene expression data over generations of PBS control (black lines) or treatment with 100nM of cetuximab (red lines). Patterns included a pattern reflecting technical artifacts between untreated controls at time 0 and subsequent generations (pattern 4) and a flat pattern for highly expressed genes (pattern 5), excluded from analysis in main figures. Heatmap of gene expression values for PatternMarker genes identified for all of these patterns. Rows were colored according to which CoGAPS pattern the PatternMarker statistic assigned each gene, and sorted by the PatternMarker statistic.

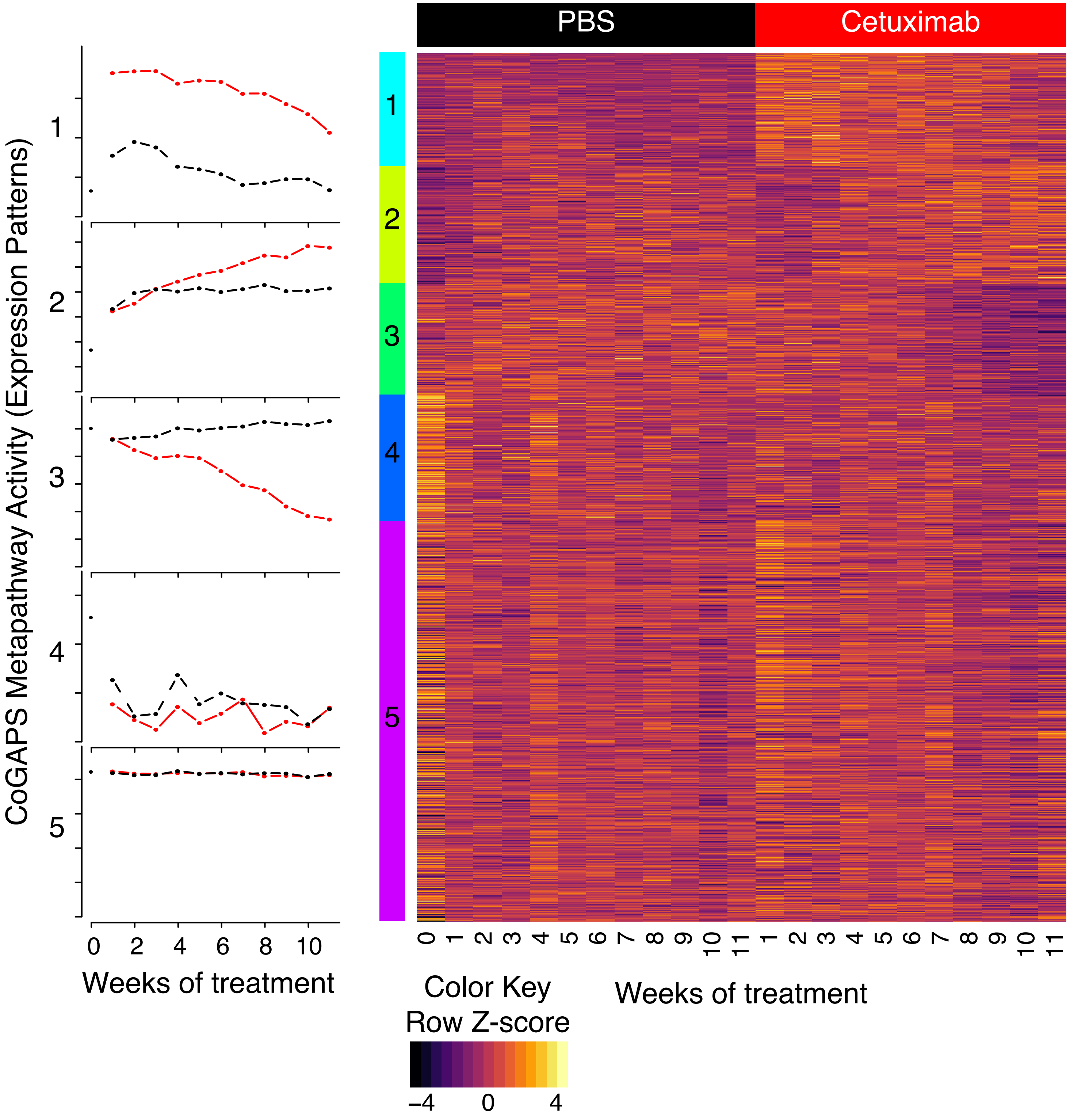

Figure S6 - Heatmap of gene set analysis scores for targets of transcription factors in the EGFR network, targets of the AP-2alpha transcription factors associated with cetuximab response, and cetuximab resistance signatures in CoGAPS patterns. A score of 100 indicated upregulation of the targets with a p-value of 0 and -100 downregulation with p-values of 0. Matrix elements with a star indicated p-values below 0.05 for either up or down-regulation of the gene set. Gene expression heatmap was colored on a red-green scale where as the gene set statistics heatmap was colored on a blue-red scale, with values indicated in the respective color keys.

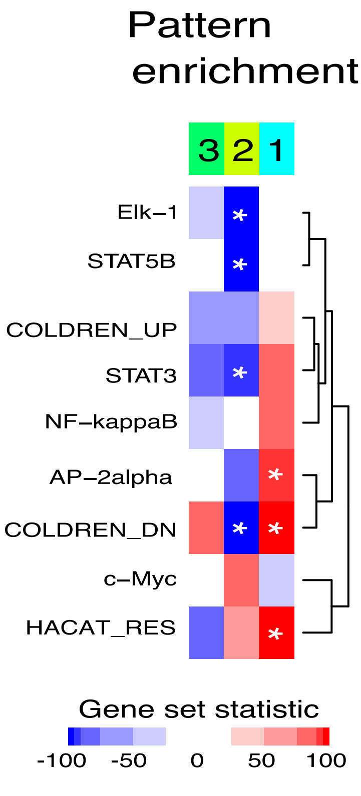

Figure S7 - Heatmaps of Pearson correlation coefficients between CoGAPS gene expression and DNA methylation patterns. A. Heatmap of Pearson correlation coefficients between CoGAPS gene expression and DNA methylation patterns. Row colors for expression patterns match the colors for patterns in Figure 2,3. The column colors for methylation patterns are selected to match the color of the corresponding expression pattern with maximum anti-correlation. B. As in A for CoGAPS gene weights (meta-pathway values) corresponding to patterns in DNA methylation (columns) and gene expression (rows).

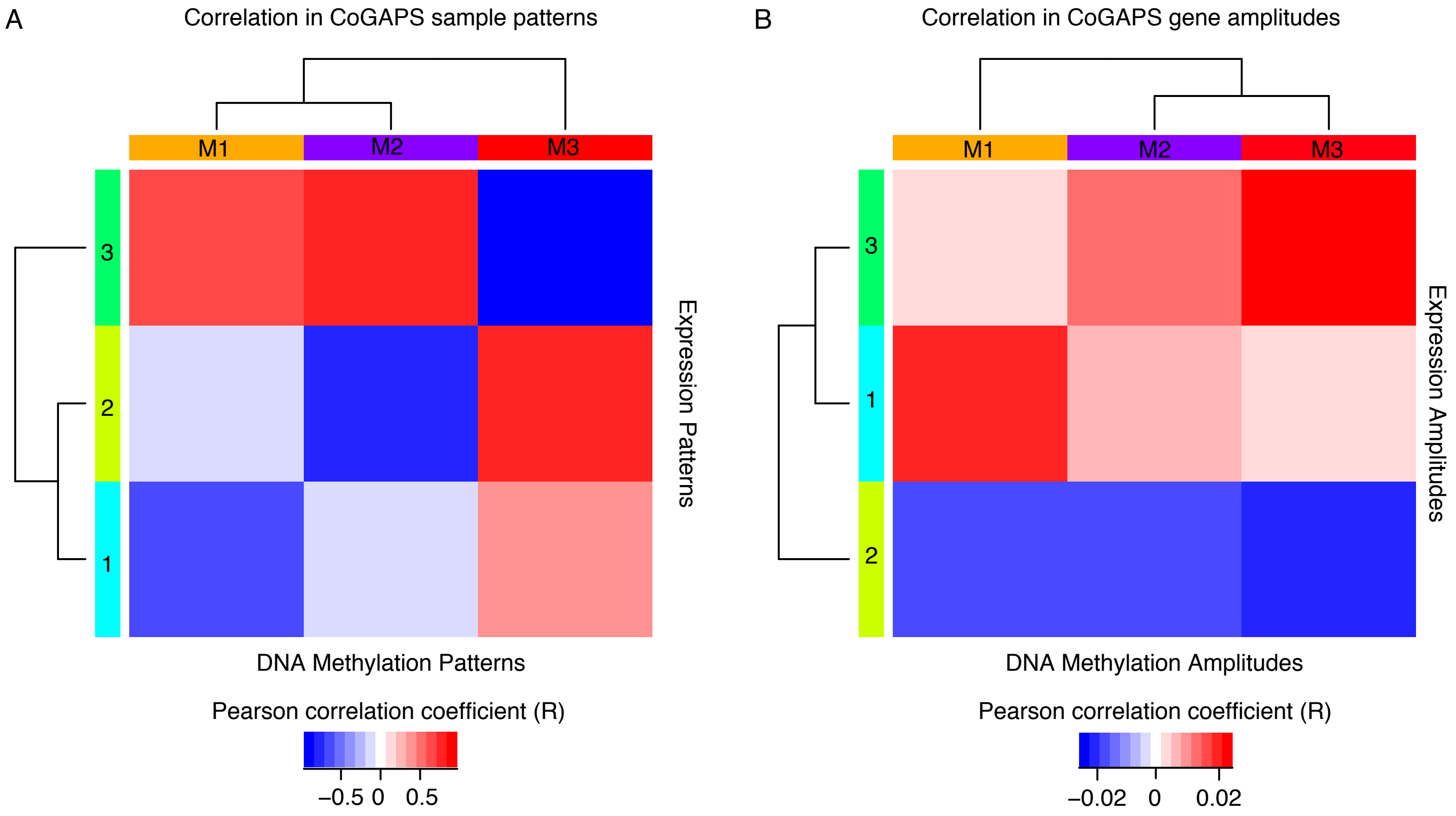

Figure S8 - Gene expression heatmap for the time course experiment vs. single cell resistant clones experiment. Heatmap of gene expression values for 11 generations of SCC25 cells treated with PBS as control (black columns labeled PBS) and with 100nM of cetuximab (red columns labeled cetuximab) to acquire resistance and gene expression data from independent, stable cetuximab resistant clones in absence of cetuximab treatment (CTX resistant clones).

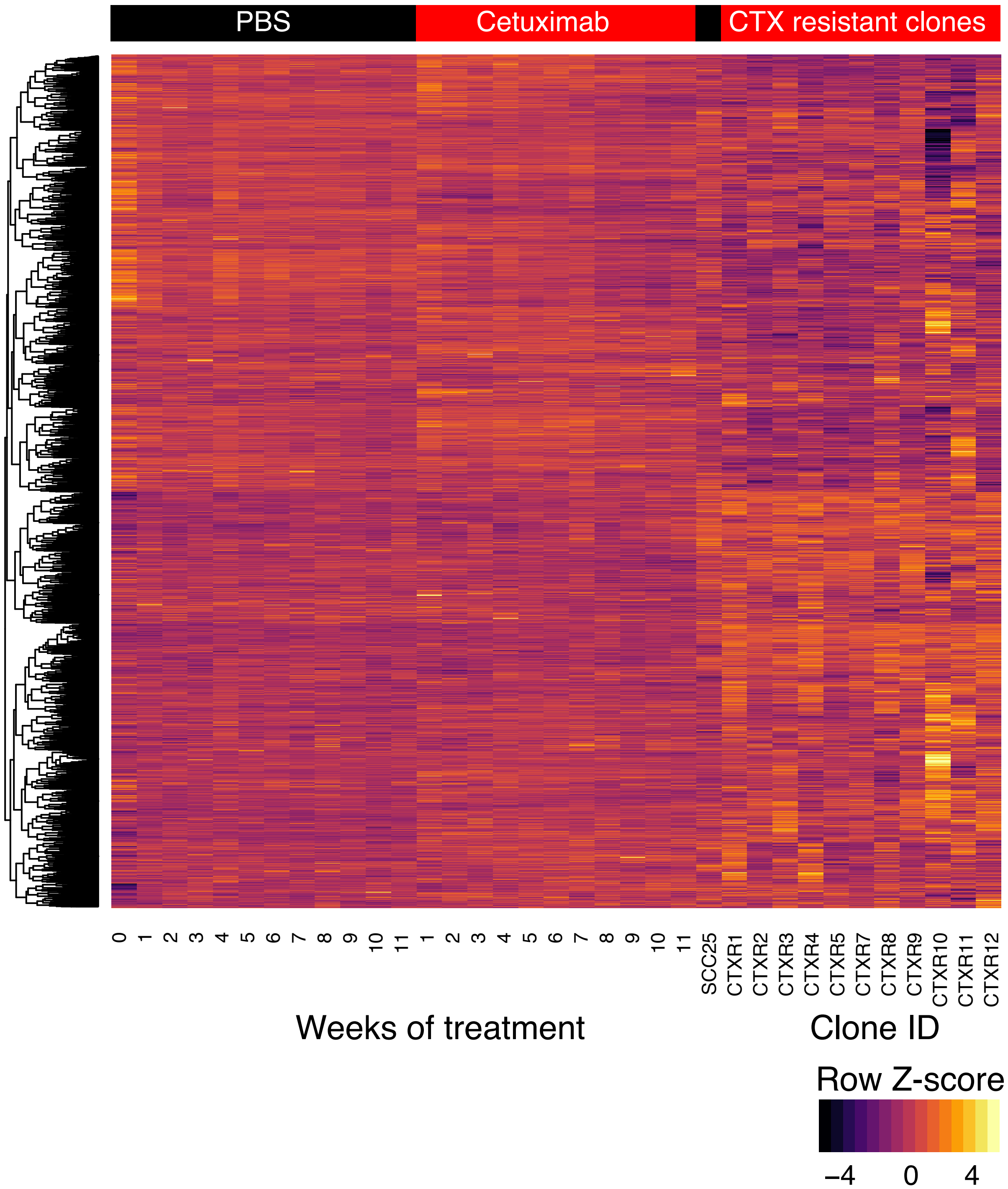

Figure S9 - DNA methylation heatmap for the time course experiment vs. single cell resistant clones experiment Heatmap of DNA methylation values for 11 generations of SCC25 cells treated with PBS as control (black columns labeled PBS) and with 100nM of cetuximab (red columns labeled cetuximab) to acquire resistance and gene expression data from independent, stable cetuximab resistant clones in absence of cetuximab treatment (CTX resistant clones).

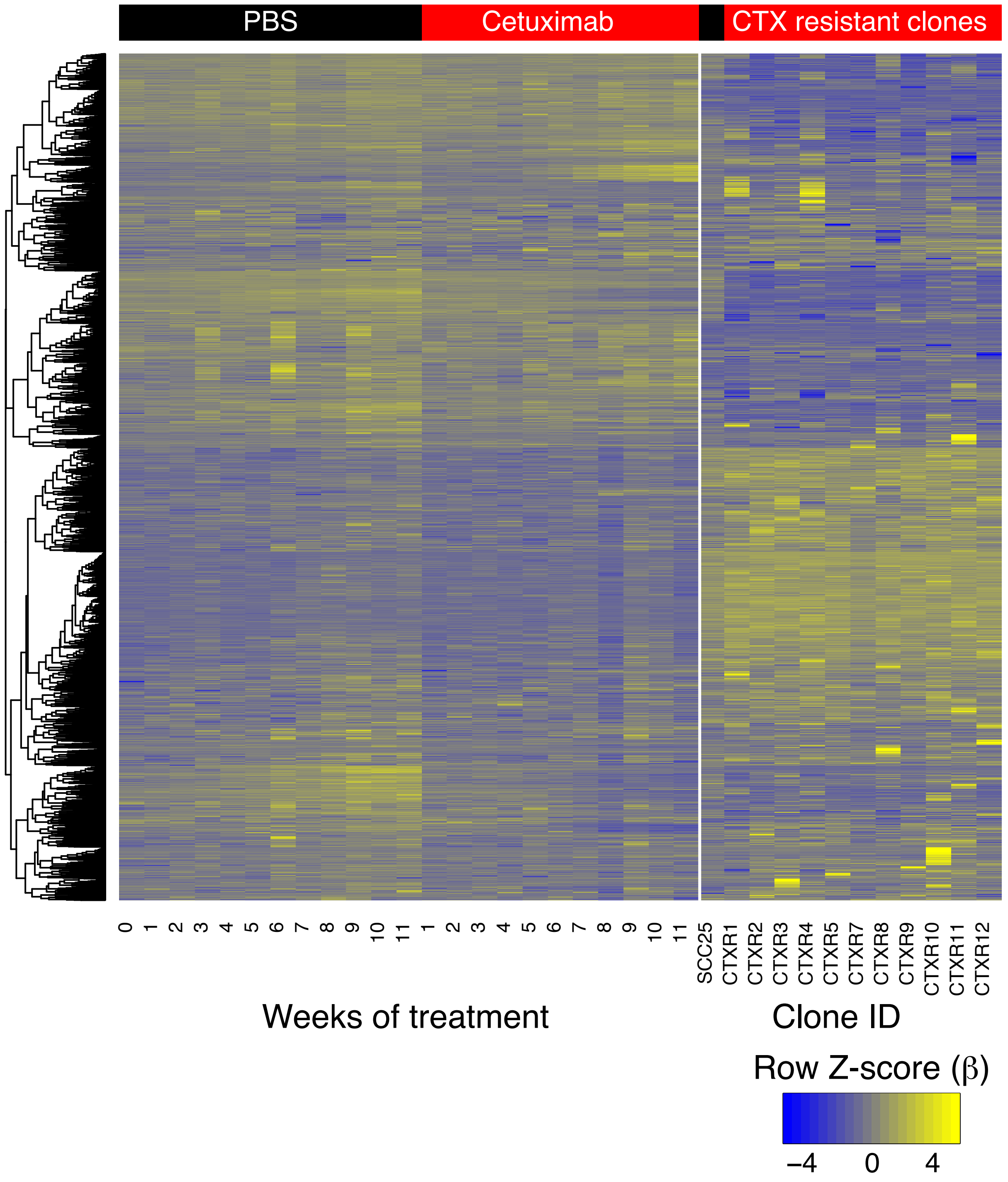

Figure S10 - Microscopy images of cetuximab single cell resistant clones (CTXR).

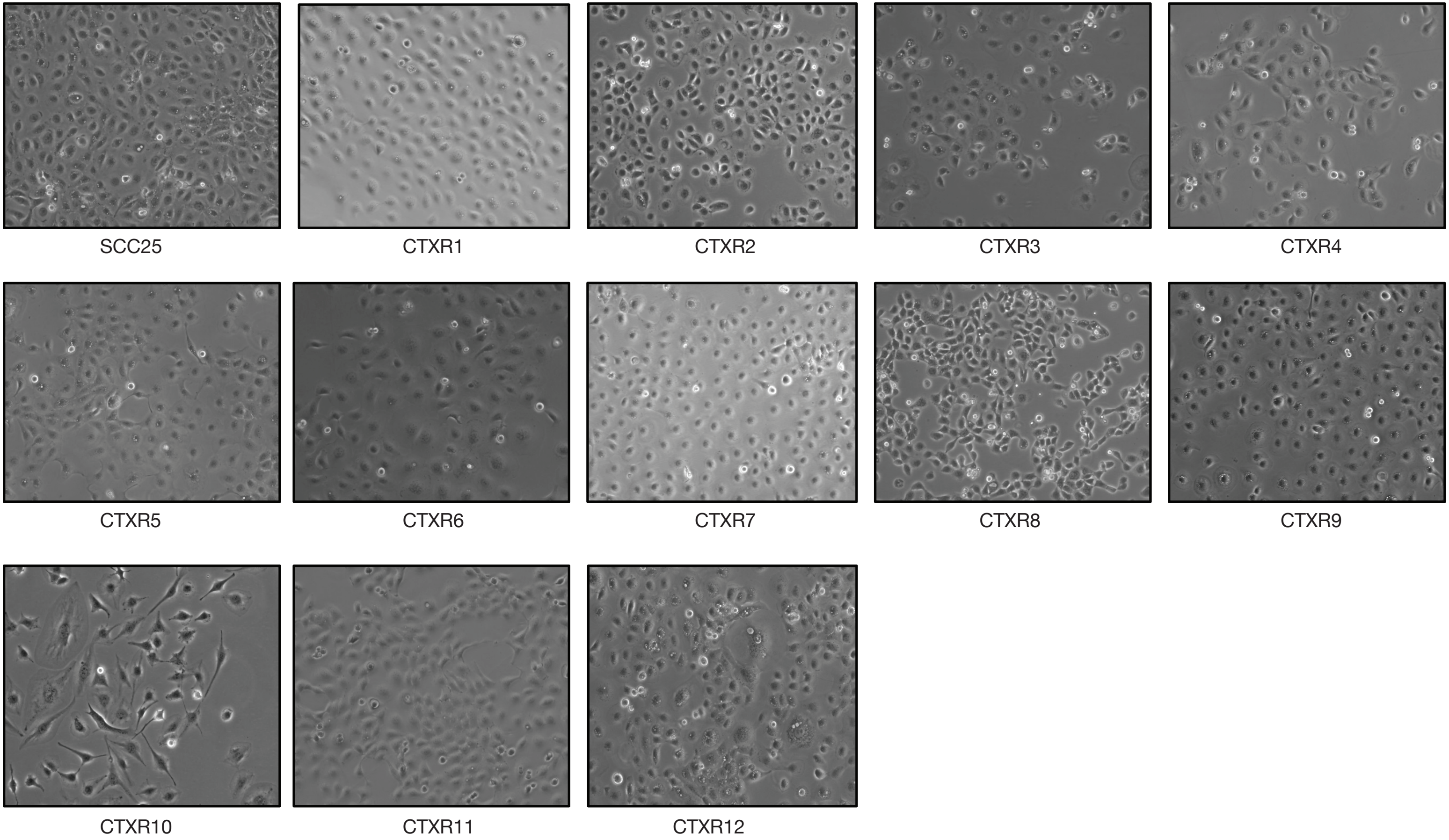

Figure S11 - Epigenetically regulated pattern marker genes associated with resistance presenting significant anti-correlation between gene expression and DNA methylation in the cetuximab single cell resistant clones (CTXR).

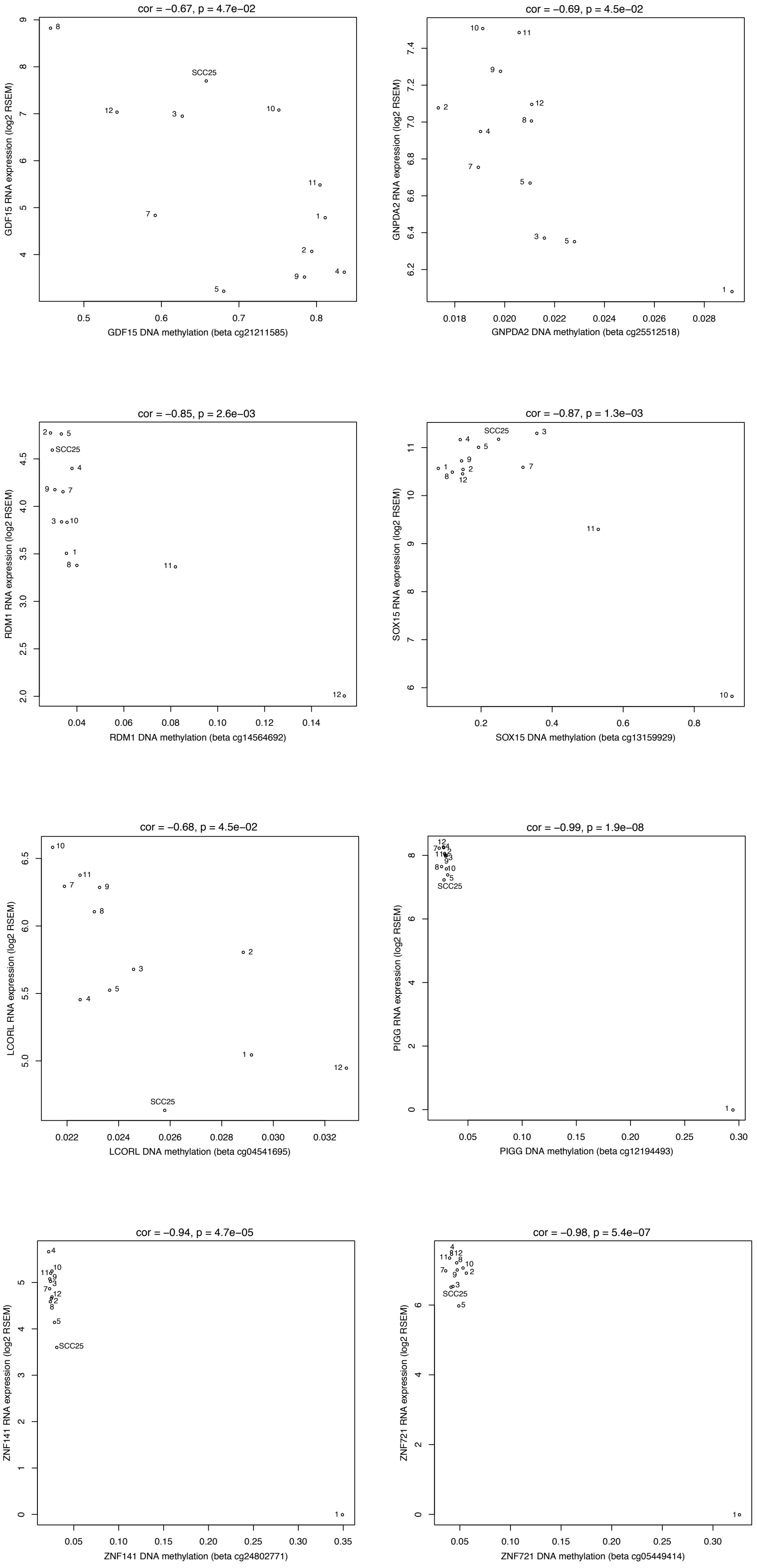

Figure S12 - Proliferation assay of cetuximab resistant single cell clones (CTXR). Cell proliferation assay using AlamarBlue (Invitrogen, Carlsbad, CA) to compare proliferation rates under different concentrations of cetuximab in the resistant single cell clones (CTXR4, 7, 10 and 11) and the parental SCC25 cell line to confirm resistance when treated with different concentrations of cetuximab.

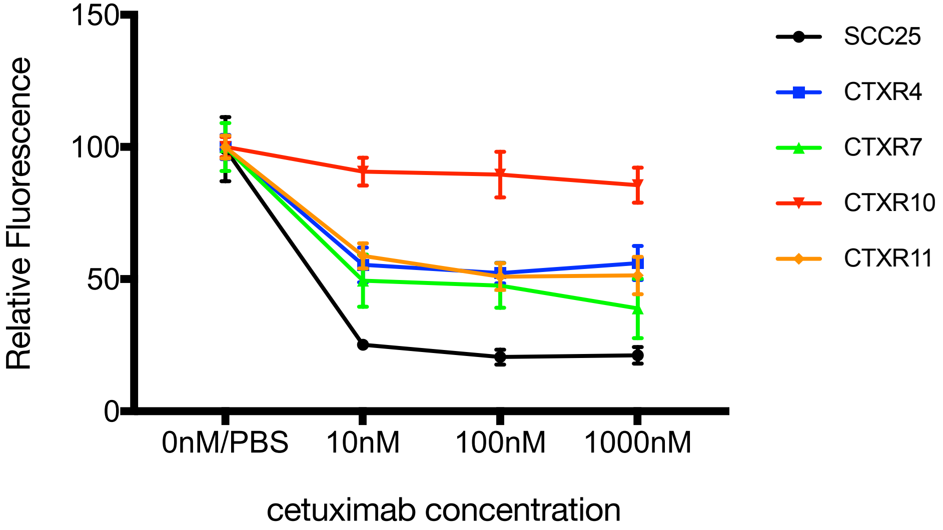

Figure S13 - Bar plot of *FGFR1* expression in human tumor samples pre (black) and post (red) treatment with cetuximab.

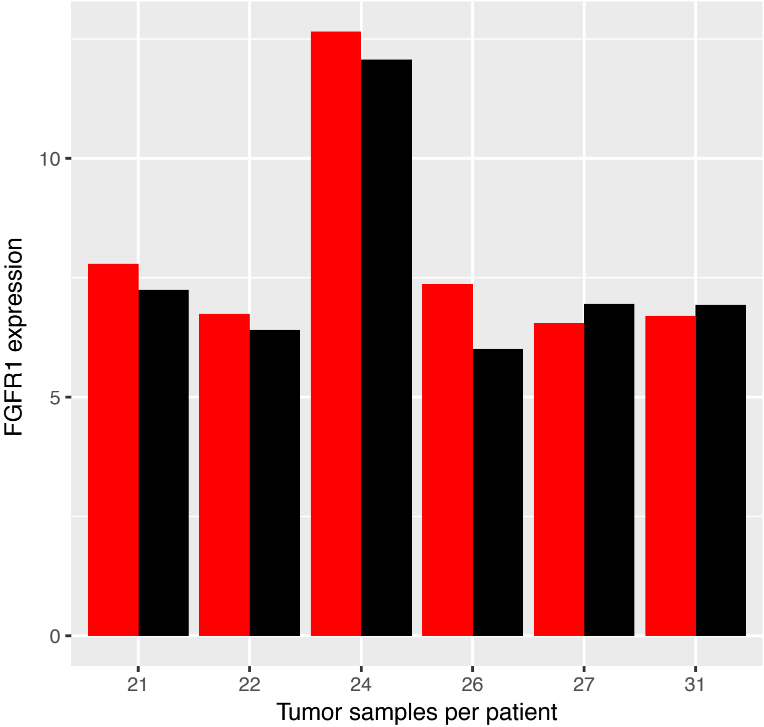

Figure S14 - Heatmap of *FGF* family genes methylation (A) and expression (B) in time course data for treated and control samples.

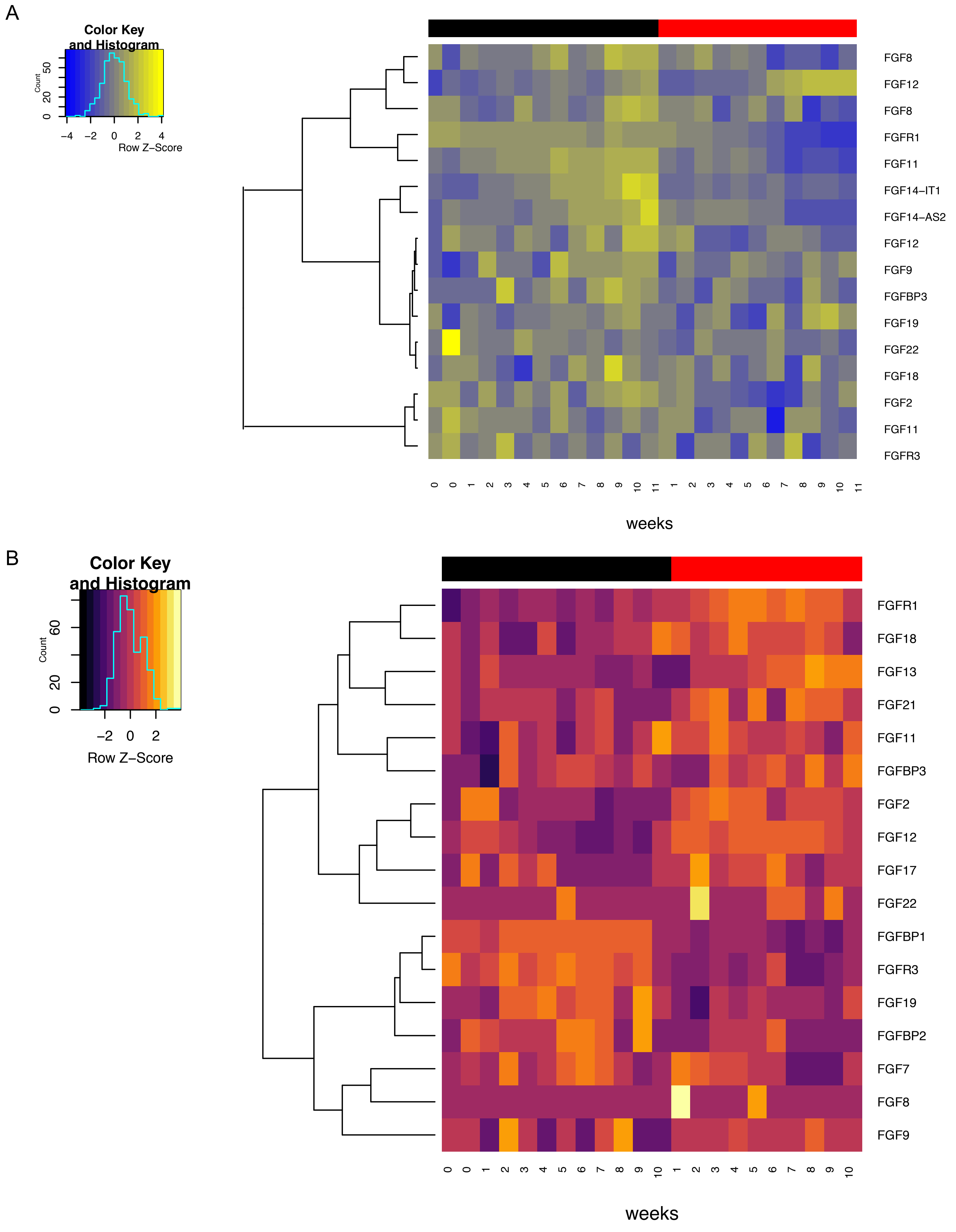

Supplement: Supplementary file 1 — Supplemental Figures S1 - S14. Figure S1 - Time course approach to induce resistance to cetuximab and measure gene expression and DNA methylation changes; Figure S2 - Anchorage-independent growth of cetuximab generation 10 (CTX-G10); Figure S3 - Heatmap and hierarchical clustering of gene expression values in 11 generations of SCC25 cells treated with PBS as control (black columns) and with 100 nM of cetuximab (red columns) to acquire resistance.; Figure S4 - Time course gene expression comparison to previously known gene signatures of resistance to EGFR inhibitors; Figure S5 - Expected gene expression values for genes in each CoGAPS pattern inferred from gene expression data over generations of PBS control (black lines) or treatment with 100 nM of cetuximab (red lines); Figure S6 - Heatmap of gene set analysis scores for targets of transcription factors in theEGFR network, targets of the AP-2alpha transcription factors associated with cetuximabresponse, and cetuximab resistance signatures in CoGAPS patterns; Figure S7 - Heatmaps of Pearson correlation coefficients between CoGAPS gene expression and DNA methylation patterns; Figure S8 - Gene expression heatmap for the time course experiment vs. single cell resistant clones experiment; Figure S9 - DNA methylation heatmap for the time course experiment vs. single cell resistant clones experiment; Figure S10 - Microscopy images of cetuximab single cell resistant clones (CTXR); Figure S11 - Epigenetically regulated pattern marker genes associated with resistance presenting significant anti-correlation between gene expression and DNA methylation in thecetuximab single cell resistant clones (CTXR); Figure S12 - Proliferation assay of cetuximab resistant single cell clones (CTXR); Figure S13 - Bar plot of FGFR1 expression in human tumor samples pre (black) and post (red) treatment with cetuximab; Figure S14 - Heatmap of FGF family genes methylation (A) and expression (B) in time coursedata for treated and control samples. [file 13073_2018_545_MOESM1_ESM.pdf]
